# 驱动基因阳性肺癌脑转移患者的靶向治疗进展

**DOI:** 10.3779/j.issn.1009-3419.2019.11.06

**Published:** 2019-11-20

**Authors:** 汶倩 李, 日兰 白, 久嵬 崔

**Affiliations:** 130021 长春，吉林大学第一医院肿瘤中心 Cancer Center, The First Hospital of Jilin University, Changchun 130021, China

**Keywords:** 肺肿瘤, 脑转移, 驱动基因, 靶向治疗, Lung neoplasms, Brain metastasis, Driver gene mutations, Targeted therapy

## Abstract

脑是晚期肺癌转移最常见的部位，驱动基因阳性者脑转移发病率更高，肺癌脑转移患者预后较差，且不同治疗方式的选择影响肺癌脑转移患者的病程转归及预后。近年来，随着精准医学的发展，针对肺癌脑转移，尤其是具有特殊靶点的驱动基因阳性患者治疗方式的选择越来越成为人们的研究热点，并逐步取得进展。本文主要阐述了肺癌脑转移治疗存在的挑战，系统综述了驱动基因阳性肺癌脑转移的靶向治疗进展，以期在精准医学时代下，指导此类患者临床实践中个体化精准治疗方案的抉择。

## Abstract (en)

Brain is the most common site of lung cancer metastasis, and the incidenceis are higher if patients have driver gene mutation. Patients with brain metastasis have a poor prognosis; further, different treatment methods affect the disease status and prognosis. In recent years, with the development of precision medicine, gradual progress has been made in treatments for lung cancer patients with brain metastasis, especially for those with driver gene mutations. This review first highlights the challenges of brain metastasis treatments, and then summarizes the research progress regarding targeted therapies for patients with driver gene mutation-positive lung cancer and brain metastasis. This review could help guide clinical decision making for individualized treatment in daily clinical practice.

原发性肺癌是我国发病率及死亡率最高的恶性肿瘤，脑是肺癌转移最常见的部位，晚期非小细胞肺癌（non-small cell lung cancer, NSCLC）脑转移率高达44%，其中驱动基因阳性者脑转移发生率为31.4%，高于阴性者（19.7%）^[1, 2]^。肺癌脑转移严重影响患者的生存期及生活质量（quality of life, QoL），脑膜转移自然病程为4周-6周，积极治疗者的中位生存时间可延长至4个月-8个月，全脑放疗联合靶向治疗者可进一步延长至11个月-12个月^[3, 4]^。脑实质转移自然病程为1个月-2个月，积极治疗者生存期可达11个月-24个月^[5]^。不同治疗方式的选择可不同程度影响肺癌脑转移患者病程转归和预后。本文主要综述了驱动基因阳性肺癌脑转移患者靶向治疗的进展及相关问题。

## 1 脑转移为肺癌治疗带来挑战

脑转移为肺癌的治疗带来挑战：一方面，血脑屏障是中枢神经系统的天然屏障，在保护大脑不受有毒物质侵害的同时却限制了药物的可及性^[6, 7]^，另一方面，不同类型脑转移（脑实质转移和脑膜转移）诊断方式的选择影响其诊断率、治疗选择及预后。增强磁共振成像（magnetic resonance imaging, MRI）是发现脑实质转移病灶的首选影像检查^[8]^，但其诊断脑膜转移的灵敏度仅为76%，其中弥漫型软脑膜转移影像表现为阴性，而脑脊液检测对于脑膜转移敏感性及特异性较高^[4, 9, 10]^。

驱动基因阳性肺癌脑转移患者的诊断和治疗面临着进一步的挑战：首先，脑转移病灶与原发病灶存在基因异质性，研究^[6]^表明53%的脑转移瘤基因突变与原发病灶不一致，且在治疗中可出现获得性突变，产生耐药和疾病进展，为精准靶向药物的选择、治疗过程中动态监测耐药突变带来挑战。其次，尽管靶向治疗可在一定程度上改善预后，但受血脑屏障限制，脑脊液中药物浓度低于血浆浓度，且不同靶向治疗药物的血脑屏障穿透性存在差异，影响治疗疗效。

由此可见，驱动基因阳性肺癌脑转移发病率高，且由于治疗药物的血脑屏障穿透性受限、诊断差异大、转移灶存在基因异质性等，使得其预后较差，因此临床实践中应更加积极的关注其治疗抉择及预后发展。

## 2 驱动基因阳性肺癌脑转移的治疗进展

肺癌驱动基因主要包括表皮生长因子受体酪氨酸激酶（epidermal growth factor receptor, *EGFR*）（突变率为19%-21%）和间变性淋巴瘤激酶融合基因（anaplastic lymphoma kinase, *ALK*）（突变率为4%-8%）及其他少见基因类型。目前探索常见驱动基因类型靶向治疗的临床试验相对较多，肺癌脑转移患者相关研究数据较为丰富，因此本文主要阐述了*EGFR*、*ALK*突变的靶向治疗研究进展^[7, 11]^。

药物能否应用于临床主要取决于大规模随机对照临床试验的结果，而考虑到脑转移所带来的无法预期的毒性、低疗效及低生存率，脑转移患者通常被排除于临床试验外，尤其是有症状或无法控制的脑转移患者，既往数据多来源于回顾性研究或小规模前瞻性研究^[12]^。有研究总结了目前临床试验对脑转移患者的纳入与排除情况，结果表明1/2-2/3的临床试验及14%-19%的新药研究试验排除了脑转移患者。而在纳入脑转移患者的临床试验中，41%的试验将纳入标准限定为接受治疗且病情稳定者。这些结果不可避免会导致临床试验结果的偏倚，影响其对临床实践的指导意义。因此，临床试验应尽量纳入脑转移患者以保证其结果的普适性^[9]^。表 1总结了目前肺癌脑转移临床试验的数据来源情况。

**1:** 肺癌脑转移患者临床试验数据来源 Data sources of clinical trials for lung cancer patients with brain metastasis

Study	Year	Treatment	Outcome	Citation
Clinical trials for patients with brain metastasis				
BRAIN	2017	Icotinib *vs* WBRT	mPFS: 6.8 mo *vs* 3.4 mo, imPFS: 10.0 mo *vs* 4.8 mo	[36]
CTONG-0803	2012	Erlotinib	ORR: 58.3%; mPFS: 15.2 mo; imPFS: 10.1 mo;	[15]
Iuchi	2013	Gefitinib	ORR: 87.8%; mPFS: 14.5 mo; mOS: 21.9 mo	[14]
Park	2012	Erlotinib or gefitinib	DCR: 93%; mPFS: 6.6 mo; mOS: 15.9 mo	[13]
William	2016	EGFR-TKI+SRS or WBRT；WBRT+EGFR-TKI；SRS+EGFR-TKI	mOS: 19.4 mo *vs* 29.9 mo *vs* 58.4 mo	[43]
William	2017	EGFR-TKI+SRS or WBRT；WBRT+EGFR-TKI；SRS+EGFR-TKI	mOS: 25 mo *vs* 30 mo *vs* 46 mo	[42]
BLOOM	2017	AZD3759	1^st^-line: ORR: 65%, iORR: 83% 2^nd^ line: ORR: 28%	[28]
Subgroup analysis for patients with brain metastasis				
ALEX	2017	Alectinib *vs* crizotinib	mPFS: 34.8 mo *vs* 10.9 mo, mPFS*: 27.7 mo *vs* 7.4 mo	[30]
ASCEND-4	2017	Ceritinib *vs* platinum-based chemotherapy	mPFS: 16.6 mo *vs* 8.1 mo mPFS*: 10.7 mo *vs* 6.7 mo	[41]
AURA3	2016	Osimertinib *vs* platinum-pemetrexed	ORR*: 71% *vs* 31%, mPFS*: 10.1 mo *vs* 4.4 mo, imPFS*: 8.5 mo *vs* 4.2 mo	[39]
FLAURA	2017	Osimertinib *vs* standard EGFR-TKI	mPFS: 18.9 mo *vs* 10.2 mo, mPFS*: 15.2 mo *vs* 9.6 mo	[27]
J-ALEX	2017	Alectinib *vs* crizotinib	mPFS: 34.1 mo *vs* 10.2 mo	[29]
LUX-Lung 3 & 6	2015	Afatinib *vs* cisplatin-based chemotherapy	mPFS*: 8.2 mo *vs* 5.4 mo, ORR*: 70% *vs* 20%	[37, 38]
NCT01970865	2018	Lorlatinib	ORR: 90%, ORR*: 66.7%	[31]
NCT01449461	2016	Brigatinib	mPFS: 15.6 mo	[32]
PROFILE 1005 or 1007	2015	Crizotinib	1^st^ line: DCR: 63%, iDCR: 56%, mPFS: 7 mo; 2^nd^ line: DCR: 65%, iDCR: 2%, mPFS: 13.2 mo	[16-18]
PROFILE 1014	2014	Crizotinib *vs* chemotherapy	ORR: 74% *vs* 45%, mPFS: 10.9 mo *vs* 7 mo, ORR*: 77% *vs* 28%, mPFS*: 9 mo *vs* 4 mo	[40]
ALTA	2017	Brigatinib	ORR: 54%, mPFS: 12.9 mo	[45]
ASCEND-5	2017	Ceritinib *vs* chemotherapy	mPFS: 5.4 mo *vs* 1.6 mo	[46]
NCT02737501	2018	Brigatinib *vs* crizotinib	ORR: 71% *vs* 60%, iORR: 78% *vs* 29%	[47]
NCT01801111	2015	Alectinib	ORR: 50%, mPFS: 8.9 mo	[48]
NCT01871805	2016	Alectinib	ORR: 48%, mPFS: 8.1 mo	[49]
NCT01964157	2017	Ceritinib	ORR: 62%, mPFS: 9.3 mo, mOS: 24 mo	[50]
NCT01336634	2016	Dabrafenib+trametinib	ORR: 63.2%, mPFS: 9.7 mo	[34]
WBRT: whole brain radiotherapy; EGFR-TKI: epidermal growth factor receptor-tyrosine kinase inhibitor; SRS: stereotactic radiotherapy; ORR: objective response rate; mPFS: median progression-free survival; mOS: median overall survival; DCR: disease control rate; *: subgroup analysis for brain metastasis lung cancer patients.				

### 2.1 靶向治疗研究进展

#### 2.1.1 靶向治疗药物的探索

##### 2.1.1.1 常规靶向治疗药物在驱动基因阳性肺癌脑转移患者中的治疗疗效

靶向治疗可使*EGFR*驱动基因阳性者获益。Park等^[13]^在一项Ⅱ期临床试验中发现，对于晚期*EGFR*突变的肺癌脑转移患者，一线应用EGFR抑制剂（EGFR-tyrosine kinase inhibitor, EGFR-TKI）治疗（吉非替尼250 mg，每天1次或厄洛替尼150 mg，每天1次）部分缓解率达83%，疾病控制率（disease control rate, DCR）达93%，中位无病生存期（median progression-free survival, mPFS）为6.6个月，中位总生存期（median overall survival, mOS）达15.9个月。另一项针对*EGFR*突变的肺腺癌脑转移患者的Ⅱ期临床试验显示EGFR-TKI治疗（吉非替尼250 mg，每天1次）时，客观缓解率（objective response rate, ORR）达87.8%，mPFS为14.5个月，mOS为21.9个月^[14]^。在TKI的二线应用中，Ⅱ期临床试验CTONG-0803研究纳入了一线含铂双药化疗失败的伴*EGFR*突变或组织类型为腺癌的无症状肺癌脑转移患者，给予厄洛替尼150 mg，每天1次，结果表明mPFS为9.7个月，mOS为18.9个月，其中，*EGFR*突变阳性患者的mPFS为15.2个月，颅内mPFS为10.1个月，ORR为58.3%，最常见不良反应为皮疹，且超过90%的不良反应为1/2级^[15]^。而对于*ALK*突变的晚期肺癌脑转移患者，靶向治疗亦改善了预后。回顾性PROFILE 1005/1007研究纳入了*ALK*重排的晚期肺癌患者，给予克唑替尼250 mg，每天2次，在无症状脑转移亚组分析中，初治患者DCR达63%，颅内DCR为56%，中位颅内进展时间为7个月。而对于复治患者，全身DCR为65%，颅内DCR为62%，中位颅内进展时间达13.2个月^[16-18]^。综上，靶向治疗可使*EGFR*及*ALK*驱动基因突变阳性的肺癌脑转移患者获益，但应用标准治疗量时，药物血脑屏障穿透性不佳，如吉非替尼平均血脑屏障透过率为1.1%-1.4%，厄洛替尼为2.8%-5.1%，脑脊液中药物浓度低于外周血中浓度，颅内病灶控制不佳，影响疗效，因此如何增加脑脊液中药物浓度是脑转移患者面临的重要问题^[19, 20]^。

##### 2.1.1.2 大剂量冲击疗法在驱动基因阳性肺癌脑转移患者中的治疗疗效

增加靶向药物剂量，给予脉冲式治疗可提高脑脊液中药物浓度，提升疗效，但其副作用不容忽视^[19, 21]^。研究^[19, 22, 23]^表明，当吉非替尼增量至1, 000 mg，每天1次，厄洛替尼可增量至600 mg每天1次或1, 000 mg-1, 500 mg，每周1次，克唑替尼可增量至750 mg-1, 000 mg，每天1次，患者仍可耐受药物副作用。一项回顾性分析探索了大剂量厄洛替尼与标准剂量EGFR-TKI在难治性EGFR阳性肺癌脑膜转移患者中的应用，结果提示大剂量靶向药物的应用一定程度改善了mOS（6.2个月*vs* 5.9个月），但无统计学意义（*P*=0.94）。大剂量药物治疗组无严重不良反应发生，且缓解了神经症状^[24]^。此外，有报道隔日应用克唑替尼1, 000 mg每天1次可提高难治性ALK阳性肺癌脑转移患者的中枢神经系统反应率，颅内进展时间达6个月，且无严重不良反应发生^[23]^。

大剂量冲击疗法可在一定程度上控制脑转移灶，作为复治脑转移患者的备选方案，应用时应注意副反应及个体化治疗。大剂量冲击疗法相关临床试验较少，目前数据主要基于回顾性研究及个案报道，尚未获得循证医学证据，故仍需大规模前瞻性临床试验数据进一步指导用药。

##### 2.1.1.3 新一代靶向药物在驱动基因阳性肺癌脑转移患者中的治疗疗效

研发血脑屏障穿透力更强的新型药物可增加脑脊液中药物浓度，新一代靶向药物血脑屏障穿透力强，奥西替尼血脑屏障透过率超过50%，新药AZD3759可达100%^[7, 25, 26]^。随机、双盲Ⅲ期FLAURA研究对比了一线应用三代EGFR-TKI奥西替尼（80 mg，每天1次）与标准EGFR-TKI（吉非替尼250 mg，每天1次或厄洛替尼150 mg，每天1次）在*EGFR*突变肺癌患者中的疗效，结果证实奥西替尼可延长无病生存期（progression-free survival, PFS），总体人群mPFS分别为18.9个月*vs* 10.2个月，在脑转移亚组分析中，mPFS分别为15.2个月*vs* 9.6个月，结果均有统计学意义，不良反应在两组中无明显差异，因此奥西替尼可作为此类患者一线治疗选择方案^[27]^。Ⅰ期BLOOM研究探索了三代靶向治疗新药AZD3759在晚期*EGFR*突变的肺癌脑转移患者中的临床效果及不良反应发生率，结果提示其在脑脊液中的浓度接近于血清浓度，证实了良好的血脑屏障穿透性。对于初治患者，ORR为65%，颅内ORR为83%。而对于复治患者，ORR为28%。当AZD3759剂量为200 mg，每天2次时，可获得良好疗效及可耐受的不良反应^[28]^。

对于ALK阳性的肺癌脑转移患者，新一代靶向药物如阿来替尼（Alectinib）、劳拉替尼（Lorlatinib）等药物的血脑屏障透过率优于克唑替尼，Ⅲ期临床试验J-ALEX对比了阿来替尼（300 mg，每天2次）与克唑替尼（250 mg，每天2次）在*ALK*突变阳性、初治或接受过一线化疗的患者中的疗效，结果提示阿来替尼相比克唑替尼，可延长mPFS（34.1个月*vs* 10.2个月），并降低严重不良反应的发生率（26% *vs* 52%），对于无症状脑转移患者，亚组分析显示阿来替尼对颅内疾病控制亦高于对照组^[29]^。Ⅲ期临床试验ALEX研究了ALK阳性的肺癌患者，一线应用阿来替尼（600 mg，每天2次）与克唑替尼（250 mg，每天2次）的疗效比较，结果显示阿来替尼可延长无进展率（68.4% *vs* 48.7%，mPFS 34.8个月*vs* 10.9个月），在脑转移亚组分析中，阿来替尼降低脑转移病灶进展率（9.4% *vs* 41.4%，mPFS 27.7个月*vs* 7.4个月），进一步证实了相比克唑替尼，阿来替尼可更好地控制脑转移病灶^[30]^。Ⅱ期临床试验NCT01970865研究提示，劳拉替尼可提升晚期ALK/ROS1阳性患者的ORR，综合对比不同线治疗，发现在总人群中ORR为90%，在脑转移亚组分析中，ORR为66.7%，即劳拉替尼也可作为*ALK*突变阳性肺癌脑转移患者的有效治疗方案^[31]^。Ⅰ期/Ⅱ期临床试验NCT01449461探索了布格替尼（Brigatinib）在ALK阳性肺癌患者中的疗效，其中对于可测量脑转移病灶患者，ORR为53%，不可测量脑转移病灶患者ORR为35%，颅内mPFS为15.6个月^[32]^。

新型靶向治疗药物血脑屏障穿透力更高，覆盖突变类型更广，临床试验表明新型靶向药物可有效控制转移灶、延缓疾病进展，为肺癌脑转移患者提供了新的治疗选择及方案，未来应进一步逐步开展针对性的大型临床研究。

##### 2.1.1.4 少见类型基因突变的靶向治疗探索

尽管少见基因突变类型在肺癌脑转移患者中表达率低，但其相应靶向治疗不容忽视，*BRAF*基因抑制剂维罗非尼（Vemurafenib）可改善*BRAF*-V600E突变肺癌脑转移患者预后^[33]^，Ⅱ期临床研究显示，达拉菲尼（Dabrafenib）联合曲美替尼（Trametinib）治疗二线*BRAF*-V600E突变晚期肺癌患者，ORR达63.2%，mPFS为9.7个月^[34]^。对于ROS1阳性患者，Ⅱ期临床试验NCT01970865研究表明劳拉替尼可提升其疗效^[31]^。RET抑制剂凡德他尼（Vandetanib）联合用药可减轻*RET*基因重排肺癌脑转移患者的颅内转移瘤负荷^[35]^。目前针对少见驱动基因类型的靶向药物正在研发，临床研究表明其在肺癌脑转移患者的治疗中初显成效，但尚缺乏大规模临床试验证实其疗效。

#### 2.1.2 靶向治疗对比常规化疗及放疗

##### 2.1.2.1 针对EGFR阳性肺癌脑转移患者

对于EGFR阳性肺癌脑转移患者，Ⅲ期临床试验BRAIN研究评估了埃克替尼与全脑放射治疗（whole brain radiotherapy, WBRT）联合化疗的疗效比较，入组人群为初治*EGFR*突变且脑转移灶≥3个的肺癌患者，结果提示埃克替尼可延长PFS，总体mPFS为6.8个月*vs* 3.4个月，颅内mPFS为10.0个月*vs* 4.8个月，同时，可减少严重不良反应的发生率（8% *vs* 38%）^[36]^。LUX-LUNG3、6比较了在晚期一线*EGFR*突变肺腺癌患者中阿法替尼（Afatinib）与含铂双药化疗的疗效，在脑转移患者亚组分析中发现，阿法替尼可延缓疾病进展（mPFS 8.2个月*vs* 5.4个月），提升ORR（70% *vs* 20%）^[37, 38]^。Ⅲ期AURA3研究显示，对于一线EGFR-TKI治疗失败后的T790M阳性*EGFR*突变的无症状脑转移晚期NSCLC患者，应用奥西替尼相比含铂双药化疗可改善患者PFS（mPFS 10.1个月*vs* 4.4个月，颅内mPFS 8.5个月*vs* 4.2个月）及ORR（71% *vs* 31%），同时降低了不良反应发生率（严重不良反应发生率23% *vs* 47%）^[39]^。综上，对于EGFR阳性肺癌脑转移患者，靶向治疗相比传统疗法改善了患者的预后，显示出了优势。

##### 2.1.2.2 针对ALK阳性肺癌脑转移患者

对于*ALK*突变NSCLC患者，Ⅲ期临床试验PROFILE 1014研究提示一线克唑替尼相比含铂化疗可改善其预后（ORR：74% *vs* 45%，mPFS：10.9个月*vs* 7个月），在脑转移亚组中，该优势（ORR：77% *vs* 28%，mPFS：9个月*vs* 4个月）亦具有统计学意义（*P* < 0.05）^[40]^。ASCEND-4研究对比了塞瑞替尼（Ceritinib）与含铂化疗在一线*ALK*重排非鳞状NSCLC患者中的应用，该Ⅲ期临床试验结果提示塞瑞替尼可提升PFS（mPFS：16.6个月 *vs* 8.1个月），在脑转移亚组分析中，塞瑞替尼同样显示出良好的疗效（mPFS：10.7个月*vs* 6.7个月）^[41]^。可见，相比传统疗法，靶向治疗亦改善了*ALK*突变肺癌脑转移患者的预后。

### 2.2 局部治疗联合全身治疗及顺序

对于肺癌脑转移患者，尤其驱动基因阳性者，靶向治疗可有效控制颅内疾病，延缓局部治疗以减少其副作用，改善患者预后。但对于有明显的临床症状、靶向治疗获益不佳的脑转移患者。局部治疗可一定程度改善颅内DCR，同时增加血脑屏障的通透性，提高药物的可及性及疗效。而靶向治疗对放疗同样有一定程度的增敏作用，可提升其局部控制率。因此，如何有效联合全身治疗与局部治疗，使患者获得最佳疗效，仍需进一步探究。

有研究^[42, 43]^进行回顾性分析探索放疗与靶向治疗应用顺序与疗效，对比立体定向放射治疗（stereotactic radiotherapy, SRT）序贯EGFR-TKI、WBRT序贯EGFR-TKI、EGFR-TKI序贯颅内放疗在*EGFR*突变阳性肺癌脑转移患者中的疗效，结果显示放疗序贯靶向治疗疗效较好，尤其SRT序贯EGFR-TKI组，可延长mOS（SRT序贯EGFT-TKI 58.4个月，WBRT序贯EGFT-TKI 29.9个月，EGFT-TKI序贯放疗19.4个月），有效延缓WBRT的神经毒性。但该回顾性分析研究也存在局限性：各组间患者基线临床特征分布不均，治疗方式的选择并非随机，此外缺少对神经症状的评估，使其不能对治疗方案进行准确全面评估。因此，局部治疗联合全身治疗方案的有效实施仍需大规模前瞻性临床试验结果证实。

由此可见，靶向治疗与常规放化疗对比中显现明显优势，单药或与局部治疗联合均可改善驱动基因阳性患者疗效。对于携带*EGFR*、*ALK*敏感突变的患者首选靶向治疗，而驱动基因阴性的患者可行全身化疗或免疫治疗，同时根据病情联合局部治疗；对于无症状脑转移患者，可首先行全身治疗，而有症状、颅外病灶稳定的脑转移患者，应积极给予局部治疗，若转移灶数目 < 3个，可选择手术治疗、SRT或SRT联合WBRT；若转移灶≥3个，可行WBRT或SRT^[5, 11]^。

## 3 展望

驱动基因阳性肺癌已进入精准治疗时代，但仍有许多问题亟需解决。首先，精准检测是精准治疗的先决条件。近年来，随着液体活检技术的不断开发，在诊断、监测疾病及治疗疗效方面取得一定进展。研究显示，通过液体活检对循环肿瘤DNA（circulating tumor DNA, ctDNA）进行检测有助于动态监测肿瘤基因突变及治疗疗效，进而指导临床决策及药物选择。研究^[9, 44]^表明，对于脑转移患者，脑脊液ctDNA水平与颅内肿瘤负荷成正比，可通过其动态监测肿瘤，更准确、敏感地反映颅内病灶的分子特征及基因表达情况，从而有效预测药物治疗疗效和疾病预后，在推动肺癌脑转移的个体化精准诊治方面具有重大意义。临床实践中，应不断探究精准液体活检技术手段，进一步提高脑脊液ctDNA检测的灵敏度及特异性，同时应不断开发探索其他新型全身性和颅脑局部疾病预测生物标志物，通过与ctDNA互补、联合动态监测病灶基因突变表达情况及靶向治疗药物的疗效，以期早于影像学有效预测疾病治疗反应性与预后转归，为患者筛选、更换最佳治疗方案，提供个体化精准治疗选择。其次，随着分子生物学研究的深入，新的驱动基因突变位点及获得性耐药突变位点不断被发现，精准高效、血脑屏障渗透性佳、毒性低的药物相继涌现；同时应进一步开展针对脑转移患者的大规模Ⅲ期临床随机对照试验，纳入不同状态的患者以减少研究中可能存在的偏倚，使药物及治疗方案的选择更具有可信度，促进精准、高效的肺癌靶向治疗模式进一步开展。最后，联合治疗为肺癌脑转移患者带来曙光，应进一步探究局部治疗与全身靶向治疗的有效联合方案，通过大规模随机临床试验明确其方式选择及治疗次序；此外，随着免疫治疗的兴起，探索靶向药物与免疫治疗、抗血管生成药物治疗方案的联合应用亦为治疗带来新的希望，期待未来在联合治疗中取得新的突破。

尽管肺癌脑转移治疗任重而道远，但目前驱动基因指导下的分子靶向治疗正逐步取得进展。在临床实践中，应权衡患者的整体情况及局部放疗所致的不良预后权衡使用，根据患者局部及全身情况综合考虑，选择最有效且不良反应最低的个体化精准治疗方案。
